# Supplementary material for: Seeing our 3D world while only viewing contour-drawings
Source: PLoS One. 2021 Jan 22;16(1):e0242581. doi: 10.1371/journal.pone.0242581 (PMC7822326; doi:10.1371/journal.pone.0242581)
Supplement: Attachment — Submitted filename: Response to Reviewers.docx [file pone.0242581.s002.docx]

Dear colleagues,

We appreciate you for reviewing our manuscript and suggestions you made. We were delighted to see that both of the reviewers are mostly satisfied with our last revision. We also thank the reviewers for understanding our situation. All the suggestions are addressed point by point below with line numbers in the revised manuscript. All the revisions are in red with balloon comments in the revised manuscript “Revised Manuscript with Track Changes”.

## Reviewer #1:

***R1a.*** *The study may have benefited from a Bayesian analysis to support the claims more strongly.*

Thank you very much for this suggestion. Note that the results of the experiments reported in this study were analyzed by using multi-way repeated-measure ANOVA but Bayesian alternatives of multi-way repeated-measure ANOVA is still under discussion (see Nathoo & Masson, 2016 for discussion). Instead, we added information of confidence intervals based on the t-distribution in the manuscript (l. 186-187, l. 238-240, l. 290-296; see Francis, 2017 for comparison between the conventional t-test and its Bayes alternative). We believe it provides better quantitative information about our results.

## Reviewer #2:

***R2a.*** *However, in the Appendix the authors should add a row to each table in order to add the average performances of the experiments with the AR device to simplify the comparison (I think that the “See Figs. 5, 8, 11 for comparison” is not enough). Moreover, they should add a short comment about the comparison. I think that this point (at least) is important to improve the paper.*

Thank you very much for this suggestion. We revised Table 1 (l. 366-371) to show average performance under the contour-drawing and grayscale-image conditions in the main experiments.

We also added a short paragraph discussing the difference between Appendix and the experiments reported in the main text of the manuscript.

## References

Francis, G. (2017). Equivalent statistics and data interpretation. Behavior research methods, 49(4), 1524-1538.

Nathoo, F. S., & Masson, M. E. (2016). Bayesian alternatives to null-hypothesis significance testing for repeated-measures designs. *Journal of Mathematical Psychology*, *72*, 144-157.
